# Interactive effects of predation risk and conspecific density on the nutrient stoichiometry of prey

**DOI:** 10.1002/ece3.1740

**Published:** 2015-10-06

**Authors:** Rafael D. Guariento, Luciana S. Carneiro, Jaqueiuto S. Jorge, Angélica N. Borges, Francisco A. Esteves, Adriano Caliman

**Affiliations:** ^1^ Ecology LaboratoryCCBSUniversidade Federal do Mato Grosso do SulCampo GrandeMSBrazil; ^2^ Department of EcologyUniversidade Federal do Rio Grande do NorteCEP 59072‐970NatalRNBrazil; ^3^ Department of Botany and ZoologyUniversidade Federal do Rio Grande do NorteCEP 59072‐970NatalRNBrazil; ^4^ Department of EcologyUniversidade Federal do Rio de JaneiroRio de JaneiroRJBrazil

**Keywords:** Ecology of stress, non‐lethal effects, nutrient balance, predation risk, stress physiology

## Abstract

The mere presence of predators (i.e., predation risk) can alter consumer physiology by restricting food intake and inducing stress, which can ultimately affect prey‐mediated ecosystem processes such as nutrient cycling. However, many environmental factors, including conspecific density, can mediate the perception of risk by prey. Prey conspecific density has been defined as a fundamental feature that modulates perceived risk. In this study, we tested the effects of predation risk on prey nutrient stoichiometry (body and excretion). Using a constant predation risk, we also tested the effects of varying conspecific densities on prey responses to predation risk. To answer these questions, we conducted a mesocosm experiment using caged predators (*Belostoma* sp.), and small bullfrog tadpoles (*Lithobates catesbeianus*) as prey. We found that *L. catesbeianus* tadpoles adjust their body nutrient stoichiometry in response to predation risk, which is affected by conspecific density. We also found that the prey exhibited strong morphological responses to predation risk (i.e., an increase in tail muscle mass), which were positively correlated to body nitrogen content. Thus, we pose the notion that in risky situations, adaptive phenotypic responses rather than behavioral ones might partially explain why prey might have a higher nitrogen content under predation risk. In addition, the interactive roles of conspecific density and predation risk, which might result in reduced perceived risk and physiological restrictions in prey, also affected how prey stoichiometry responded to the fear of predation.

## Introduction

Organisms under predation risk can alter their resource use and metabolic demand (Guariento and Esteves 2013; Sheriff and Thaler 2014), and consequently, their nutritional budget, body nutrient composition, and excretion rates and ratios (Hawlena and Schmitz 2010a,b; Dalton and Flecker 2014). The nutrient demand of an organism is affected by the intensity of its physiological processes, which, among other factors, might depend on its level of stress (Steiner and Van Buskirk 2009; Hawlena and Schmitz 2010b). At a specific level of predation risk, an increase in prey metabolism is one of the most evident physiological traits. In the short term (i.e., minutes), such a metabolic increase ensures that the prey can be energetically able to avoid or fight its predators (Steiner and Van Buskirk 2009; Hawlena and Schmitz 2010b). On the other hand, if such a metabolic increase is maintained over the long term (i.e., days), the prey is forced to relocate energy from growth or storage to meet the metabolic energy demand. This mechanism may inhibit prey biomass production and nutrient excretion, and, in extreme cases, promote the breakdown of body proteins into glucose (i.e., gluconeogenesis) (Hawlena and Schmitz 2010a,b). Adjustments in the foraging behavior of prey related to resource choice can prevent the deleterious effects of the increase in energy demand induced by predation risk (Hawlena and Schmitz 2010a,b). Therefore, alterations in the nutrient excretion of prey in response to predation risk might affect rapid nutrient cycling and mineralization through organism ingestion and posterior excretion, while alterations in the resource choices of prey might affect slow nutrient cycling and organic matter left to the detritivore chain (Vanni 2002).

Ecological stoichiometry (ES) is a conceptual framework that analyzes the constraints and consequences of the mass balance of multiple chemical elements in consumer–resource interactions (Sterner and Elser 2002). ES provides mechanisms for understanding how imbalances between an organism and its food affect its physiology, population dynamics, and ecosystem‐level processes (Alves et al. 2010; Guariento et al. 2011a, 2011b). Consequently, it is possible to trace directly an individual prey's stoichiometry plasticity in response to predation risk, which alters the prey's nutrient body and excretion stoichiometry and can reverberate throughout ecosystem‐level processes (Sterner and Elser 2002; Schmitz 2008; Hawlena and Schmitz 2010b; Leroux et al. 2012). For example, *Melanoplus femurrubrum* grasshoppers facing spider predation risk have a greater demand for carbon (C) than control grasshoppers do, leading to changes in the grasshoppers’ diet, with consequences that affect nutrient recycling (Hawlena and Schmitz 2010a). Not many studies have investigated whether and how predation risk might directly and indirectly modify prey body and excretion nutrient stoichiometry, respectively (Dalton and Flecker 2014). The general stress paradigm (GSP) (Hawlena and Schmitz 2010a) asserts that predation risk ultimately increases the organism's energetic demand, shifting the fate of C from secondary production to maintenance and resulting in the release of excess nutrients associated with structural tissues, mostly nitrogen (N) and phosphorus (P). The rationale behind this hypothesis derives from the notion of organism threshold elementary ratio (TER) (Frost et al. 2006), which represents the elementary ratio of resources that shifts the nutrient limitation of an organism. The explicit formulation of an organism's TER of C to nutrients is

$${\text{TER}}_{C:N}=\frac{A_{N}}{{\text{GGE}}_{C}}\frac{Q_{C}}{Q_{N}}$$

where *A*_N_ represents nutrient assimilation efficiency, GGE_C_ represents the gross growth efficiency of C, and *Q*_C_ and *Q*_N_ represent the organism's body quantity of C and nutrients, respectively. GGE_C_ can be described as

$${\text{GGE}}_{C}=\frac{(I_{C}A_{C})-R}{I_{C}}$$

where *I*_C_ corresponds to the amount of ingested C, *A*_C_ represents C assimilation efficiency, and *R* represents respiration rate, or the organism's energetic demand. GGE_C_ ultimately represents the amount of ingested C that goes into the production of new tissues. Under predation risk, GGE_C_ decreases because energetic demand tends to increase (Hawlena and Schmitz 2010a). A reduction in GGE_C_ leads to an increase in TER_C:N_, which means a greater demand for C. Under predation risk, therefore, organisms experience C limitation and reduced secondary productivity. Because organisms in general lack metabolic pathways to store nutrients, the excess nutrients that were previously allocated for secondary production tend to be excreted.

However, predictions based on GSP have recently been contested. Costello and Michel (2013) and Dalton and Flecker (2014) have provided different perspectives regarding the effects of predation risk on prey TER_C:N_, and therefore, nutrient allocation and excretion in the presence of predators. Using tadpoles as their model system, Costello and Michel (2013) claimed that due to adaptive morphological responses to predation risk, tadpoles tend to reduce N excretion under predation risk due to an increased demand for N in order to build larger tail muscles. This evidence supports many previous observations of additional responses by prey, beyond physiological stress, in the presence of predators (Preisser and Bolnick 2008). Prey might increase refuge use (Lima and Dill 1990), produce spines (Tollrian 1995), or change their shape (Guariento et al. 2015) in the presence of predators, which in turn can affect body nutrient content and recycling patterns (Costello and Michel 2013; Dalton and Flecker 2014). Using guppies as a model system, Dalton and Flecker (2014) reported that guppies tended to increase N assimilation efficiency and N content as an adaptive response to a reduced quantity and quality of resources in refuges, thus sparing high‐quality molecules (i.e., proteins) from catabolism. These perspectives contradict the overall predictions of the GSP, because TER_C:N_ would have to decrease, leading to a higher demand for nutrients, and not increase, as predicted by the GSP. Therefore, physiological, behavioral, and morphological responses to predation risk are likely to interact in their influence on nutrient cycling and might overcome the changes predicted by the GSP (Costello and Michel 2013).

In this study, we aimed to test GSP predictions; however, one critical aspect of the aforementioned effects on prey phenotype is how prey assess and react to predation risk (Hettye et al. 2012). Prey organisms possess numerous sensory pathways for predator detection, such as vision, hearing, and chemical cues (Saidapur et al. 2009; Hettye et al. 2012). Prey can detect the presence of predators via direct contact or through indirect recognition of the cues released during successful or unsuccessful predator attacks on other prey individuals (Peacor 2003). According to Peacor (2003) and Van Buskirk et al. (2011), conspecific density can strongly influence prey risk assessment. Thus, prey density might weaken the risk effects on prey traits by reducing the perceived risk (Van Buskirk et al. 2011). As such, we conducted an experiment across a gradient of prey conspecific densities to address this mechanism. Our hypotheses are that (1) predation risk decreases the body N and P content of prey and, consequently, increases prey excretion rates; and (2) such effects of predator‐induced stoichiometry plasticity is mediated by prey density.

## Methods

An outdoor mesocosm experiment was conducted at the Agricultural School of Jundiai (EAJ‐UFRN), Macaíba, Rio Grande do Norte, Brazil. The experimental units were truncated, cone‐shaped fiberglass tanks (mesocosms) approximately 200 L in volume (0.74 m diameter base; 0.98 m diameter aperture; 0.53 m height). Two weeks prior to starting the experiment, the mesocosms were filled with water from an adjacent water body to introduce the natural physical and chemical conditions representative of local water bodies, as well as the local assortment of periphyton propagules. To test our hypotheses, we exposed bullfrog tadpoles (*Lithobates catesbeianus*) (~1.5 cm length; 0.015 g mean weight; 20 days old) to signals of predation risk (i.e., chemical cues) produced by adult giant water bugs (~3.0 cm length), *Belostoma* sp. One day prior to starting the experiment, tadpoles belonging to the same cohort (~10 days old) were obtained from a frog farm located in Pium, Rio Grande do Norte. The giant water bugs were collected from nearby natural temporary pools. In addition, to verify homogeneity of the experimental starting conditions among the mesocosms, we collected water samples (20 mL) from each mesocosm prior to beginning the experiment and stored them in plastic vials. The water samples were kept under frozen storage until the nutrient (total N and P) analysis.

The experiment followed a 2 × 3 full factorial design, with two levels of predation risk (with risk/no risk) and three levels of prey conspecific densities (12, 24, and 36 ind/m^3^, or 3, 6, and 9 individuals per mesocosm, respectively). The tadpole densities were chosen to mimic the range of tadpole densities in nearby natural ponds. The treatments were replicated three times, for a total of 18 experimental mesocosms. All of the mesocosms were covered with mosquito nets to prevent oviposition and immigration by aquatic insects, predators, or competitors. Predation risk was manipulated by caging two giant water bugs in individual plastic floating cages inside each mesocosm. The cages (~10 cm in diameter and ~17 cm in length) were made of transparent PET bottles with the ends enclosed by mosquito netting, and they were attached to a small piece of polystyrene foam to raise the top of the cage 3 cm from the water surface, thus allowing the water bugs to breathe. The density of water bugs per mesocosm was based on previous studies that found several effects tied to predation risk (Guariento and Esteves 2013). The caged water bugs were fed one conspecific tadpole every other day throughout the experiment in order to maintain predation cues in the mesocosms. The tadpoles used to feed the water bugs were housed in separate mesocosms. We inspected the mesocosms daily and replaced any dead water bugs. As a result, water bug density and predator risk signals were constant throughout the experiment. The experiment lasted 19 days, a time range similar to those of previous studies in which behavioral observations were conducted (Peacor and Werner 2001) and long enough to affect tadpole stoichiometry under predation risk (Guariento and Esteves 2013).

Tadpole mortality was monitored and recorded throughout the experiment. However, the dead tadpoles were not replaced, in order to ensure that the time of exposure to risk cues was the same among the remaining tadpoles. Overall, the average mortality rates were ~15%. Using conspecific density and predation risk as predictor variables and mortality as the response variable, we found that tadpole mortality was not affected by predation risk (*P* = 0.51; GLM), conspecific density (*P* = 0.18; GLM), or their interactions (*P* = 0.69; GLM). Therefore, we considered the final tadpole density per mesocosm instead of the initial density in our statistical analysis.

We used the methods described in Schaus et al. (1997) to quantify the tadpoles’ excretion rates. After 19 experimental days, all tadpoles in each mesocosm treatment were captured and immediately placed in plastic containers filled with 150 mL of filtered (using GF/F filters) water from local water bodies that the tadpoles naturally inhabit. Water samples (20 mL) were collected from each container to quantify the initial nutrient concentrations in the water prior to adding the tadpoles. The tadpoles were incubated for 85–95 min, in accordance with the procedures of Whiles et al. (2009), after which they were removed and euthanized. Final water samples (20 mL) were filtered through Whatman GF/C filters to remove feces and other particles, stored in acid‐washed vials, and frozen until nutrient analysis. The samples were analyzed for ammonia (NH_3_) and orthophosphate (${\text{PO}}_{4}^{-3}$), using the salicylate hypochlorite method (Golterman et al. 1978) and the ammonium‐molybdate method (Strickland and Parson 1978), respectively. Mass‐specific nutrient excretion rates (Exc) were calculated as follows:

$$\text{Exc}=\left(\left[N_{\mathrm{initial}}\right]-\left[N_{\mathrm{final}}\right]\right)/VTB^{3/4}$$

where [*N*_initial_] is the initial concentration of the nutrient (NH_3_ or ${\text{PO}}_{4}^{-3}$), [*N*_final_] is the final concentration of the nutrient (NH_3_ or ${\text{PO}}_{4}^{-3}$), *V* is the container volume in liters, *T* is the incubation time in hours, and *B* is the tadpoles’ total dry‐weight biomass in milligrams. We raised the biomass to ¾ power to account for the allometric relationship between excretion and biomass (Torres and Vanni 2007).

To quantify the body nutrient stoichiometry of the tadpoles, they were dried at 60°C for a minimum of 48 h, weighed (to the nearest 0.01 g), and ground to a fine powder with a mortar and pestle. The powder samples were digested with 3% potassium persulfate to convert particulate N and P to nitrate (${\text{NO}}_{3}^{-}$) and phosphate (${\text{PO}}_{4}^{-3}$), respectively (Suzumura 2008). ${\text{NO}}_{3}^{-}$ was measured using a Total Carbon Analyzer with a nitrogen analyzer module (TOC‐V Shimadzu^®^), and ${\text{PO}}_{4}^{-3}$ was measured using the ammonium–molybdate method (Strickland and Parson 1978). N and P tissue contents were determined based on the dry weights of the samples used in the analysis and were expressed as N or P (*μ*g) per total tissue dry weight (mg). Due to limitations in the availability of the powder samples, one replicate from our 12 ind/m^3^ treatment was not carried to analysis.

Because periphyton stoichiometry (a main food item for the tadpoles in the experiment) might modify the tadpoles’ body and excretion stoichiometry (Guariento et al. 2011a) independently of the experimental treatments, we collected periphyton samples from each mesocosm just prior to beginning the experiment in order to assess the homogeneity of the N:P periphyton stoichiometry among the treatments. The periphyton samples were collected by scraping with a plastic card at two different depths, placed previously in the mesocosm in three random areas of the mesocosm walls, according to the method of Guariento et al. (2010, 2011b). The scraped periphyton were then rinsed into vials and filled up to 50 mL to create a slurry with deionized water. The total N and P concentrations in the periphyton at the beginning of the experiment were estimated using the same chemical analysis used to estimate the tadpoles’ N and P contents. The periphyton N:P ratio was not affected by the predation risk x conspecific density interaction (*F *=* *0.229; df* *= 1; *P *=* *0.639; ANOVA). We also observed no individual significant effects of predation risk (*F *=* *0.178; df* *= 1; *P *=* *0.678; ANOVA) or conspecific density (*F *=* *0.513; df* *= 1; *P *=* *0.225; ANOVA) on the N:P ratio of the periphyton growing on the walls of our experimental units.

To quantify morphological predator‐inducible prey plasticity, we sampled all individual tadpoles from every mesocosm at the end of the experiment, euthanized them, and transferred them to the laboratory. We then took dorsal and lateral photographs of each individual tadpole and used the photographs to perform digital measurements of the width and depth of the tail muscle. These measurements were used as a direct proxy for predator‐inducible morphological plasticity, and tadpole body length was used to correct for allometric effects on tail measurements due to body size differences among the tadpoles. We decided to use these morphological traits because the expression of predator‐induced phenotypes can be very specific to the predator's hunting mode (Miller et al. 2014). Belostomatide are highly versatile predators that alternate rapidly between ambush and active tactics (Cloarec 1990). Defense against active foragers such as *Belostoma* sp. (R. D. Guariento, pers. obs.), which usually capture prey with single strikes, requires that the prey shows strong swimming abilities (Teplitsky et al. 2004). A proxy of muscle mass was inferred from the product between the width and depth of the tail muscle, which represents a cross‐section of the tail muscle.

### Statistical analysis

The stoichiometry measurements of the tadpoles are intrinsically multivariate (Liess and Hillebrand 2006); they include the body (%N, %P, and N:P ratio) and excretion (NH_3_, PO_4_, and NH_3_: ${\text{PO}}_{4}^{-3}$ ratio). We first used principal component analysis (PCA) to reduce the prey's stoichiometric measurements to two main variables (Legendre and Legendre 2012), body nutrient stoichiometry and excretion nutrient stoichiometry. Body nutrient stoichiometry was represented by the main (greater explicability) PCA axis scores for %N, %P, and N:P ratio measurements. Excretion nutrient stoichiometry was represented by the main PCA axis scores for NH_3_, PO_4_, and NH_3_: ${\text{PO}}_{4}^{-3}$ ratio measurements. We used only the main PCA axis (i.e., PC 1) for both variables (i.e., body and excretion stoichiometry) because it returned a proportion of variance greater than 90% (i.e., 93% for body stoichiometry and 95% for excretion stoichiometry), thus capturing most of the correlation structure of the data. To evaluate which variables were most related to the PCA scores, we performed a linear regression model between the PCA scores and actual stoichiometric measurement values. The slope values of each variable indicate how that variable is related to the PCA scores. We then used a generalized least squares model (GLS) to evaluate the individual and interactive effects of predation risk (fixed factor) and tadpole density (covariate) on tadpole body and excretion nutrient stoichiometry.

In accordance with our objectives, a significant interaction term between predation risk and tadpole density confirms that prey density mediates the effects of predation risk on response variables. The raw values of each response variable regressed against the conspecific density of treatment, with and without predation cues, are available in the supplementary material (Appendix S1A). Despite the fact that a potential linear relationship along conspecific density (our covariate) is expected only for predators with type 1 functional responses (Peacor 2003), our maximum conspecific density was relatively low compared with similar experimental designs (Van Burskirk et al. 2011). Assuming that giant water bugs have type 2 functional responses, we believe the low conspecific density used for the present experiment ensured that our observations stayed within the linear range of a type 2 functional response (Burskirk et al. 2009). We used a linear mixed‐effects model (LME) fitted by a restricted maximum likelihood method to evaluate the individual and interactive effects of predation risk (fixed factor) and tadpole density (fixed factor) on the tail muscle mass of the tadpoles. To remove the effect of individual body length on morphological traits, thereby allowing differently sized organisms to have different relationships with predation risk and conspecific density, we included body length (random factor) as a random term in the model, thus creating a random‐intercept model (Zuur et al. 2009). We also used Spearman's rank correlation to test whether muscle mass was correlated with the percentage of tadpole N content. We used Spearman's correlation due to the nonlinear but monotonic relationship between muscle mass and body percentage of N. This nonlinear relationship is expected because muscle mass was estimated from two linear measurements (~cm^2^) and percentage of N would be related to muscle volume (~cm^3^).

We used a two‐way ANOVA to test whether the total N and P concentrations in the water were statistically homogeneous among the treatments at the beginning of the experiment. No noteworthy effects were found among the experimental units (Appendix S1B).

All statistical analyses were performed using R software version 3.2. All graphics were generated using GraphPad Prism version 5.01 for Windows (GraphPad Software, San Diego, CA).

## Results

**Figure 1:**
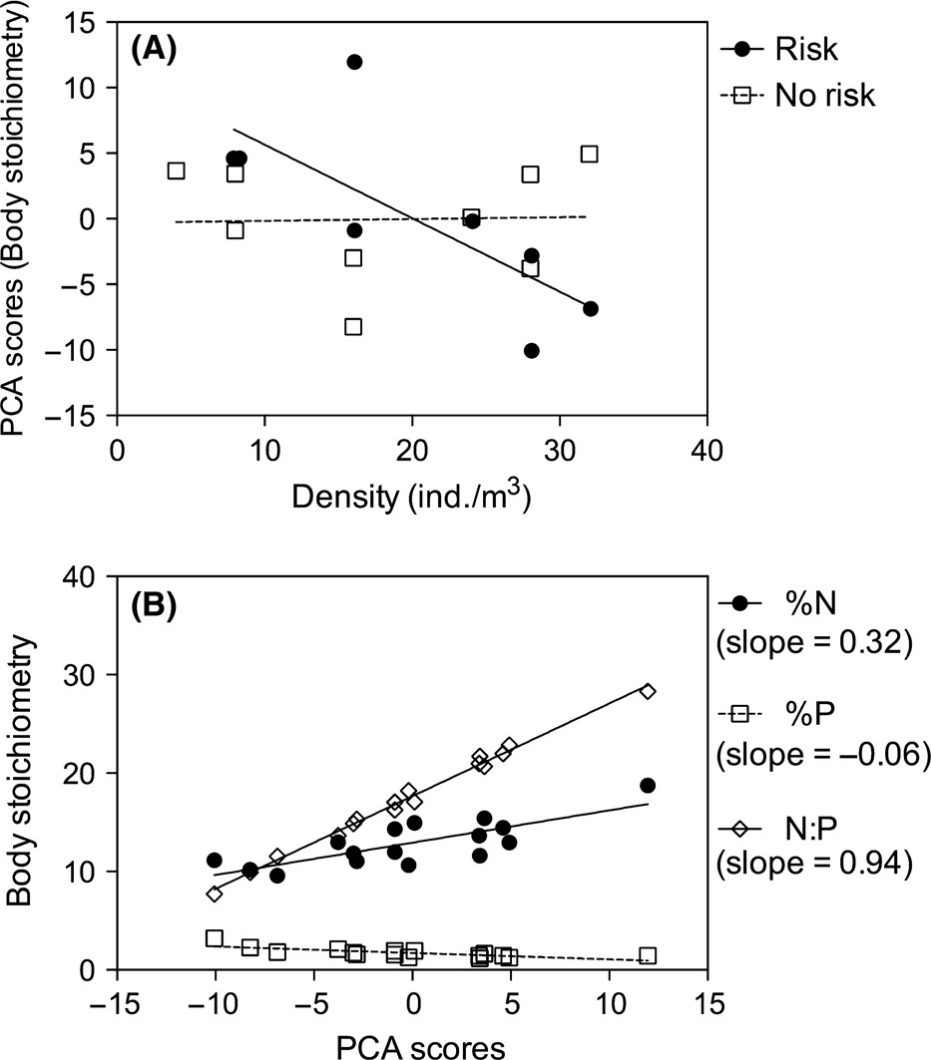
(A) Tadpoles body nutrient stoichiometry (PCA scores from %N, %P, N: P ratio measurements) regressed against conspecific final density in the presence and absence of predation risk cues. (B) Relationship between tadpole body nutrient content (N and P) and stoichiometry (N:P ratio) and PCA body stoichiometry scores. Variables with higher slope values are more related to PCA scores.

The body nutrient stoichiometry of the tadpoles was not affected significantly by conspecific density (*t *=* *0.39; df = 1; *P *=* *0.69; GLS); however, it was affected significantly by predation risk (i = 2.20; df = 1; *P *=* *0.04; GLS) and interaction between the two variables (*t *=* *‐2.21; df = 1; *P *=* *0.02; GLS) (Fig. 1A). Prey body nutrient stoichiometry (depicted by PCA scores) decreases as conspecific density increases, but only in treatments with predation risk. We observed that the N content and N:P ratio of the tadpoles were strongly related with the PCA scores (Fig. 1B‐ Appendix S1C). Therefore, a reduction in body nutrient stoichiometry primarily represents a reduction in prey body N and N:P contents.

**Figure 2:**
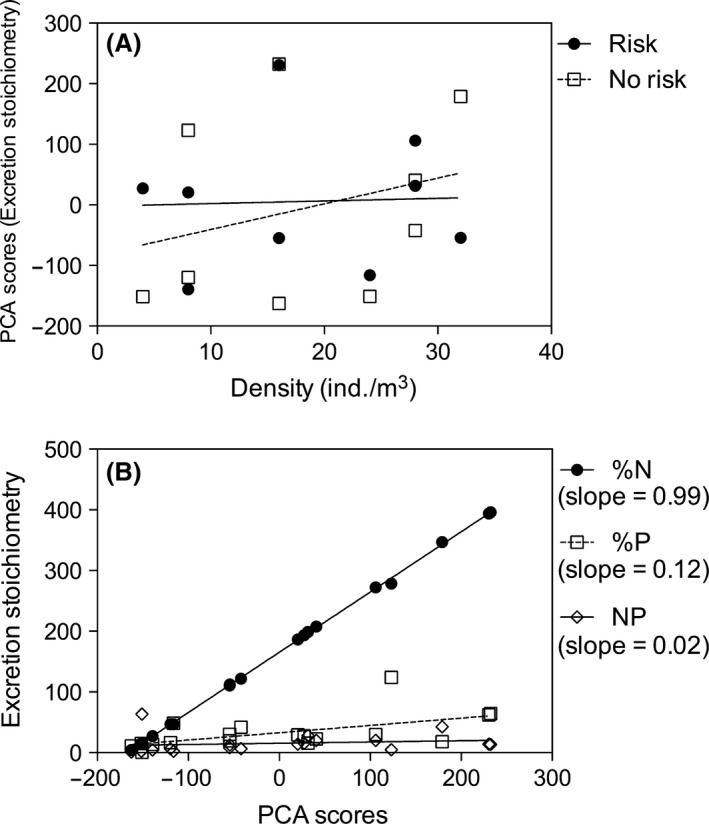
(A) Tadpoles excretion nutrient rates and ratio (PCA scores from NH_3_, PO_4_, NH_3_: ${\text{PO}}_{4}^{-3}$ ratio measurements) regressed against conspecific density in the presence and absence of predation risk cues. (B) Relationship between mass‐specific NH_3_ excretion rate, mass‐specific ${\text{PO}}_{4}^{-3}$ excretion rate, and N:P excretion ratio with PCA excretion stoichiometry scores. Variables with higher slope values are more related to PCA scores.

Different from the body stoichiometry results, the excretion nutrient stoichiometry of the tadpoles was not affected by predation risk (*t *=* *0.56; df = 1; *P *=* *0.58; GLS), prey density (*t *=* *0.86; df = 1; *P *=* *0.40; GLS), or interactions between the two variables (*t *=* *−0.54; df = 1; *P *=* *0.59; GLS) (Fig. 2A).

**Figure 3:**
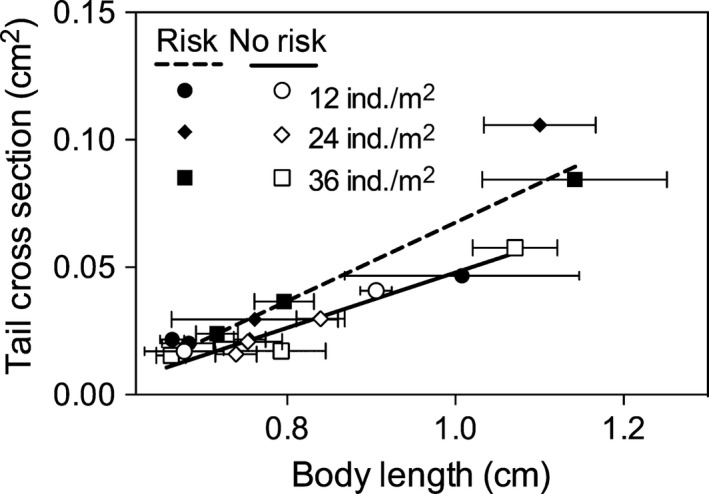
Relationship between tadpoles body N content and tadpoles tail muscle mass, depicted by the size of tail muscle cross‐section (see Methods for more details). The positive correlation was statistically significant (*P *=* *0.037; Spearman), and the solid line represents the fitted model.

Finally, predation risk affected tadpole muscle mass significantly (*t *=* *3.29; df = 38; *P *=* *0.002; LME); individuals under predation risk exhibited greater muscle mass (Appendix S1D). Further analysis also indicated that muscle mass was significantly associated with greater N body content in the tadpoles (*r *=* *0.54; *P *=* *0.037; Spearman; Fig. 3).

## Discussion

While the distinction between predation risk and actual predation has been well established in theoretical and empirical studies of predator–prey interactions (Schmitz et al. 2004), some classical examples have neglected the role of predation risk on community and ecosystem dynamics (Peckarsky et al. 2008). In addition, very few studies have evaluated the role of predation risk on the physiological traits of prey, such as elemental stoichiometry (Costello and Michel 2013; Dalton and Flecker 2014). The GSP (Hawlena and Schmitz 2010a,b) is a theoretical framework that connects stoichiometry and predation risk. However, most GSP tests have focused on species that exhibit only behavioral responses, especially related to habitat shifting. In the present study, we refuted GSP predictions and our main hypothesis, yet our results indicate that nutrient‐based predictions based on the GSP are confounded when the prey can exhibit morphological plasticity. Previous studies have also found that phenotypic responses such as reduced foraging activity (Dalton and Flecker 2014) or responses other than behavioral ones (Costello and Michel 2013) can confound GSP predictions. We believe that adaptive morphological responses in risky situations explain why the prey in treatments with predation risk had higher N contents. However, confirming our second hypothesis, the interactive role of conspecific density and predation risk, which might alter the prey's perceived risk (Peacor 2003) and induce physiological restrictions (Davenport and Chalcraft 2014), might also affect how prey stoichiometry responds to predation risk.

Traditional stoichiometric theory holds that predation does not influence prey body C:nutrient ratios, because the prey must maintain relatively tight homeostatic body C:nutrient ratios to survive and reproduce (Elser 2000). This view, however, embraces the notion that predator effects on prey are entirely consumptive. However, predation can elicit fear responses in prey, leading to physiological stress that might be manifested as increased metabolism and respiration and the synthesis of heat‐shock proteins, which together affect the prey's elemental composition (Hawlena and Schmitz 2010a,b; Hawlena et al. 2012; Leroux et al. 2012). These predictions are derived from the notion that the physiological responses of animal prey to predation risk lead to changes in nutrient use and retention. Predation risk is predicted to increase metabolism, thereby promoting protein catabolism and retaining less N in the tissues (Hawlena and Schmitz 2010a). In the present experiment, however, predation risk increased prey N content, an effect that diminished as prey density increased. Costello and Michel (2013) provided a mechanistic explanation for this unexpected pattern. They found that the body N:P ratio of tadpoles increased in the presence of predators, and their results suggest that changes in body morphology (e.g., tail muscle width) rather than behavioral defenses were most likely responsible for predator‐mediated differences in body stoichiometry. This outcome is due to the nitrogen‐rich nature of muscle mass, highlighting that strong morphological defenses might overwhelm or counteract the nutrient predictions of GSP. Dalton and Flecker (2014) also provided explanations for the pattern we observed in our study. Across a wide taxonomic spectrum, vertebrate animals faced with food restriction preferentially mobilize glycogen and lipid stores for energy production (Wang et al. 2006), thereby increasing the C:N ratio of their metabolism, reducing amino acid catabolism, and lowering the production of N waste as NH_3_ (Sinha et al. 2012; Liew et al. 2013). The adaptive significance of these changes is a sparing of the resource stores (i.e., protein) most needed for future physiological activities (McCue 2010), thereby increasing the efficiency of N retention (Kousoulaki et al. 2010; Akpınar et al. 2011).

We suggest that the increased N body content of the predator‐exposed prey in the present experiment was caused by a strong morphological response to the predators (i.e., increase in tail muscle mass), which could be corroborated by antipredator strategies that adaptively spare proteins from catabolism. While some organisms, such as grasshoppers, continue to forage when sheltering from predators, selectively choosing foods with a high C:N ratio (Hawlena and Schmitz 2010a), fish and other organisms, such as tadpoles (Guariento and Esteves 2013), have been widely shown to reduce feeding behaviors when predators are present (Peacor and Werner 2001; Wojdak and Luttbeg 2005). Therefore, it is expected that reduced feeding under predation risk and possibly, a lack of diet choice in refuges, prevent tadpoles from exhibiting the elevated metabolism and N excretion rates that would be expected based on predation risk‐induced physiological change alone (Hawlena and Schmitz 2010b).

In this study, the N body content of the tadpoles decreased and the N:P ratio increased with increasing density in treatments with predation risk. To the best of our knowledge, this is the first experiment to show this type of effect. This result might simply be due to the fact that perceived predation risk decreases as prey density increases, thereby reducing the need to invest in greater muscle mass. One explanation for this pattern is the “risk assessment” mechanism (Peacor 2003; Van Buskirk et al. 2011), suggesting that *L. catesbeianus* tadpoles incorporate prey density information into the predation risk assessment. The “risk assessment” mechanism assumes that prey investment in antipredator defenses should vary with levels of both indirect risk cues and prey density. Such a mechanism was supported previously by Van Buskirk et al. (2011) and McCoy (2007). Van Buskirk et al. (2011) reported that *Rana temporaria* tadpoles respond behaviorally to *per capita* predation risks imposed by *Aeshna cyanea* dragonfly larvae and that these responses decreased as prey conspecific density increased. McCoy (2007), in turn, reported that morphological predator‐induced changes in *Hyla chrysoscelis* were reduced in response to increased conspecific density when subjected to predation risk imposed by the *Lethocerus americanus* giant water bug.

Nevertheless, at the highest prey density, N body stoichiometry was lower than prey body stoichiometry from treatments with no predation cues. This result is unlikely if only assume the risk assessment mechanism is assumed. Prey N content in high conspecific density should at least equal the observed in treatments with no predation risk. This outcome is expected because conspecific density decreases perceived risk just until conditions are reached that are similar to zero risk. Therefore, different mechanisms might affect prey N allocation in high conspecific density when under predation risk. Dalton and Flecker (2014) also showed that cue‐exposed prey excretes less N due to the restricted consumption of food, limited by predator presence. Guariento and Esteves (2013) found similar results for P excretion when studying tadpoles under predation by belostomatids. Restricted food consumption is more likely to occur in treatments with high conspecific density due to greater intraspecific competition and in the presence of predation risk, which reduces prey activity (Guariento et al. 2014). Indeed, we observed that prey individuals were most frequently found resting at the bottom of the mesocosm and less engaged in foraging and swimming in treatments with risk (R. D. Guariento, pers. obs.). Both factors (greater chance of intraspecific competition and limited activity) might contribute to restricted food consumption and help to explain the prey N limitations observed in our study. This argument is corroborated by previous studies that found that high conspecific density can lead to physiological limitations, thus restricting antipredator responses (Davenport and Chalcraft 2014). Despite the notion that consumers are strictly homeostatic (i.e., that they do not vary their elemental composition regardless of their nutritional content), some studies have shown that this is not entirely true (Guariento et al. 2011a,b) and that stoichiometric plasticity is indeed expected due to food restriction.

Contrary to our body stoichiometric results, predation risk and prey density had no effect on the tadpoles’ excretion stoichiometry, suggesting that *L. catesbeianus* tadpoles maintained elemental excretion homeostasis in the presence of predators. This result might be related to the fact the excretion was assessed only as a potential measurement. In the present experiment, excretion was measured only once, at the end of the experiment. However, prey must integrate their phenotype; therefore, behavior and physiology are not independent (Murren 2012). Although physiological changes might have led to different molecule allocations, once a physiological defense is expressed, behavioral responses might be less useful or necessary for prey, allowing for increased or reduced foraging to offset any costs in the long term, which might have occurred in our experiment toward its end. However, body nutrient stoichiometry is not affected as quickly as excretion rate; therefore, it might have served as a much more integrative response to predation risk effects during the course of our experiment. Previous studies have corroborated the argument that the costs of inducing and reversing behavioral defenses (manifested as reducing activity or increasing refuge use) are low, which ensures a rapid response to threat (Relyea 2003). In contrast, physiological adjustments to risk, such as altering body nutrient budgets, should be much more costly to express or revert (Hammill et al. 2010; Hawlena and Schmitz 2010b).

Hawlena and Schmitz (2010a) showed that predation risk reduced (by 6%) the N content of grasshoppers, which led to an increase in the C:N ratio of the body and a concomitant increase (of 7%) in the C:N ratio of fecal debris entering the detritus chain. These changes in nutrient recycling can translate into altered rates of ecosystem function (Hawlena et al. 2012). Therefore, the effect of predation risk on prey body stoichiometry might reverberate on larger scales, especially because excretion is associated with overall prey homeostasis or food uptake. Dalton and Flecker (2014) showed that predation risk strongly reduces N excretion rates in guppies, especially due to the reduction in foraging activity. Our experiment showed that body N and N:P ratio contents of tadpoles can increase in the presence of predation risk. These changes may translate into richer detritus releasing into the detritivore chain, with further consequences to ecosystem functioning (Schmitz 2013).

Peckarsky et al. (2008) showed that many classical examples of density‐mediated effects in ecological communities reported in the ecological literature were actually behaviorally driven, highlighting the fact that individual responses might be widespread in the population, and that they might play an important role in overall ecosystem functioning. The results presented here show that *L. catesbeianus* tadpoles adjust their nutrient stoichiometry in response to predation risk and that this effect is affected by conspecific density. Our results show that the tadpoles in treatments with predation risk exhibited greater muscle mass. Greater muscle mass was also positively related to body N content. This phenotypic plasticity is generally considered adaptive, as tadpoles with greater muscle mass are better able to escape predators (Van Buskirk and Relyea 1998). Therefore, morphological responses to predation risk can confound predictions based on the GSP. Costello and Michel (2013) also challenged GSP predictions, arguing that most tests of the GSP (Hawlena and Schmitz 2010a) have focused on species that exhibit only behavioral responses to predators. Although we did measure responses that are counter to the GSP, it is not evident that the tadpoles did not exhibit a stress response. For example, we did not measure the hormones (e.g., glucocorticosteroids) that initiate the cascade of physiological responses characteristic of the GSP (Hawlena and Schmitz 2010a). Thus, we cannot say whether the stress response was initiated or whether it changed relative to the expression of prey morphological response. Very recently, the ecophysiological effects of predation risk were brought into the ecological framework (Sheriff and Thaler 2014). Physiological responses underlie many of the cost, benefit, and trade‐off decisions prey make in defensive investment and antipredator responses (Relyea and Auld 2004). Clearly, predators alter prey directly through killing and indirectly through alterations in their physiology. Our results corroborate such evidence and serve as a mechanistic explanation for the link between evolutionary processes (i.e., predator‐induced phenotypic responses) and ecosystem functioning (i.e., nutrient cycling).

## Conflict of Interest

None declared.

## Supporting information


**Appendix S1.** (A) Tadpoles body nutrient content (right panels) and excretion rates (left panels) regressed against conspecific density at the end of the experiment in the presence and absence of risk predation cues. (B) Comparison of N (Nitrogen) and P (phosphorus) concentrations and N:P ratio (mean ± SD) in water column among treatments at the beginning of the experiment. (C) Loadings (i.e., eigenvalues) and percentage of variation in data for principal components analysis of the three measures of tadpole body and excretion stoichiometry. (D) A proxy of muscle mass was inferred from the product between tail muscle width and depth, which represents a cross section of the tail muscle.
