# Supplementary material for: Interactive effects of predation risk and conspecific density on the nutrient stoichiometry of prey
Source: Ecol Evol. 2015 Oct 6;5(21):4747–56. doi: 10.1002/ece3.1740 (PMC4662331; doi:10.1002/ece3.1740)

**Interactive effects of predation risk and conspecific density on the nutrient stoichiometry of prey**

Rafael D. Guariento^1*^; Luciana S. Carneiro.^2^; Jaqueiuto S. Jorge^3^; Angélica N. Borges.^3^, Francisco A. Esteves^4^, and Adriano Caliman^2^

Running head: Predation risk and prey stoichiometry

^1^Ecology Laboratory, CCBS, Universidade Federal do Mato Grosso do Sul, Campo Grande, MS, Brazil

^2^Department of Ecology, Universidade Federal do Rio Grande do Norte, CEP 59072-970, Natal, RN, Brazil

^3^Department of Botany and Zoology, Universidade Federal do Rio Grande do Norte, CEP 59072-970, Natal, RN, Brazil

^4^ Department of Ecology, Universidade Federal do Rio de Janeiro, Rio de Janeiro, RJ, Brazil

*Corresponding author e-mail: [rafaguariento@gmail.com](mailto:rafaguariento@gmail.com)

Appendix 1A - Tadpoles body nutrient content (right panels) and excretion rates (left panels) regressed against conspecific density at the end of the experiment in the presence and absence of risk predation cues.


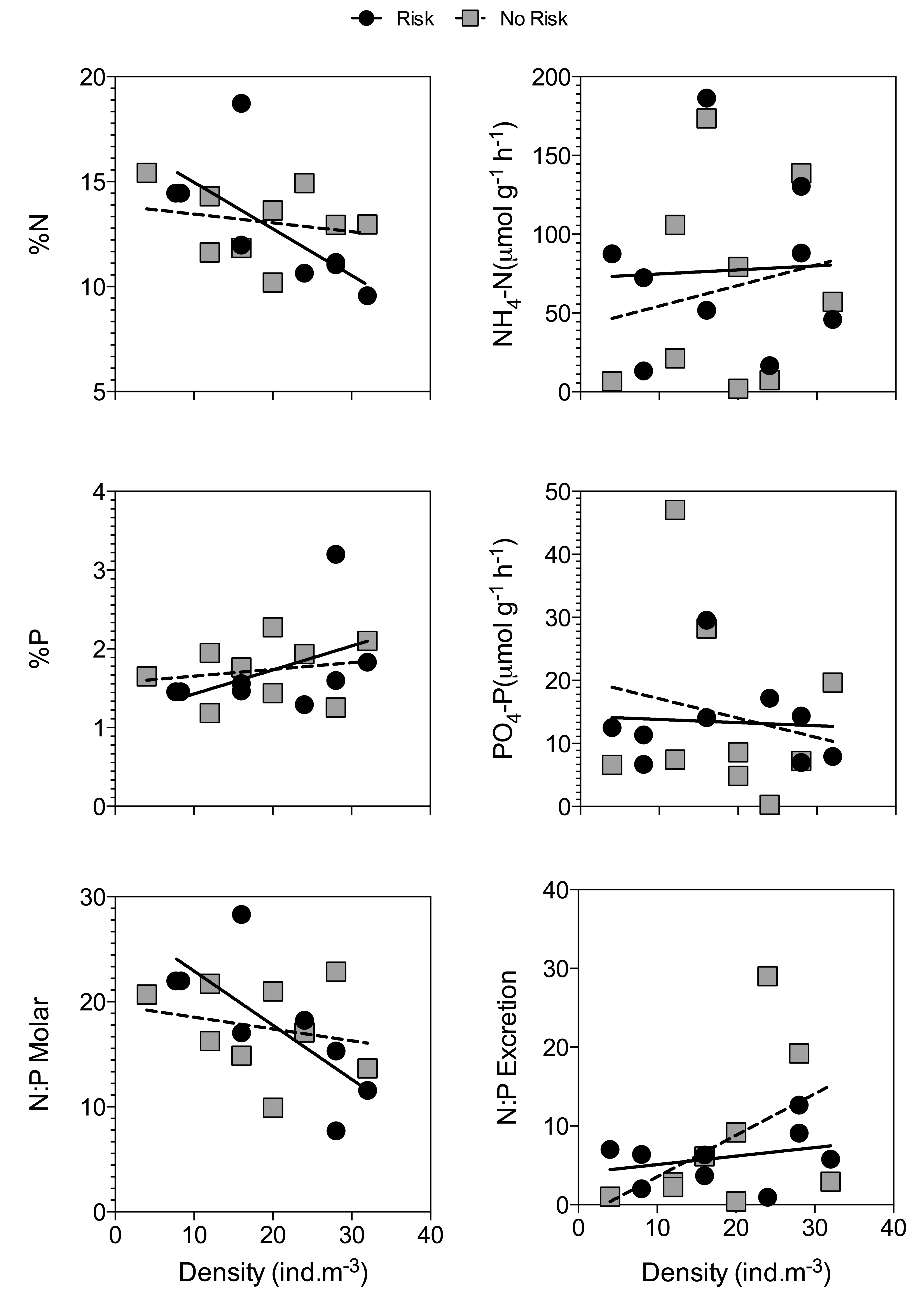


Appendix 1B – Comparison of N (Nitrogen) and P (phosphorus) concentrations and N:P ratio (mean ± SD) in water column among treatments at the beginning of the experiment. (*) Denote treatments that are significantly different among each other (Two factor ANOVA, Tukey post hoc test; *P* < 0,05).

| Tadpole initial density (ind./ m^3^) | Risk | No Risk |
| --- | --- | --- |
| [N] |  |  |
| 12 | 82.656 (±1.926) | 82.910 (±1.979) |
| 24 | 84.675 (±1.514)* | 77.785 (±4.3)* |
| 36 | 86.623 (±0.172) | 81.996 (±3. 876) |
| [P] |  |  |
| 12 | 7.40 (±0.646) | 5.3 (±14.690) |
| 24 | 7.33 (±0. 441) | 7 (±0.513) |
| 36 | 7.47 (±0.191) | 7.33 (±1,705) |
| N: P molar |  |  |
| 12 | 11.191 (± 0.645) | 20. 532 (±14.690) |
| 24 | 11.563 (±0.44) | 11.144 (±0.513) |
| 36 | 11.603 (±0.191) | 11.320 (±1.704) |

The analysis of the chemical conditions of the water column in our experimental units, showed that at the beginning of the experiment, total nitrogen (N), phosphorus (P) concentrations and their ratio (N: P ratio) among treatments (*risk* and *no risk*) were equal, except to nitrogen concentration between treatments containing 24 ind./m^3^ of prey density. In this treatment, we observed that N concentration was lower at *no risk* compared to *risk* treatments. However, the actual difference was very low and should have very little biological influence.

Appendix 1C - Loadings (i.e., eigenvalues) and percentage of variation in data for principal components analysis of the three measures of tadpole body and excretion stoichiometry. The first principal components (PC) explained 93% and 95% of the total variation in the data for tadpole body and excretion stoichiometry respectively.

|  |  | Loadings |  |
| --- | --- | --- | --- |
| Variable | PC1 (0.93%) | PC2(0.069%) | PC3(< 0.001%) |
| Body Stoichiometry |  |  |  |
| [N] | 0.32842982 | 0.9323466 | -0.1512074 |
| [P] | -0.06515121 | 0.1820685 | 0.9811251 |
| N: P molar | 0.94227871 | -0.3123794 | 0.1205402 |
|  |  |  |  |
| Excretion Stoichiometry | PC1 (0.95%) | PC2(0.036%) | PC3(0.008%) |
| [N] | 0.99254366 | 0.0999830 | 0.0697172 |
| [P] | 0.12014941 | -0.8988407 | -0.4214850 |
| N: P molar | 0.02052332 | 0.4267187 | -0.9041515 |

Appendix 1D – A proxy of muscle mass was inferred from the product between tail muscle width and depth, which represents a cross section of the tail muscle. The picture below represents the tail muscle cross sections for treatments with and without predation risk (Risk and No Risk respectively) for different conspecific densities along different individual body lengths. Predation risk positively affected tadpole muscle mass (t-value = 3.29; df = 38; p = 0.002; *Linear Mixed Effect Model*).


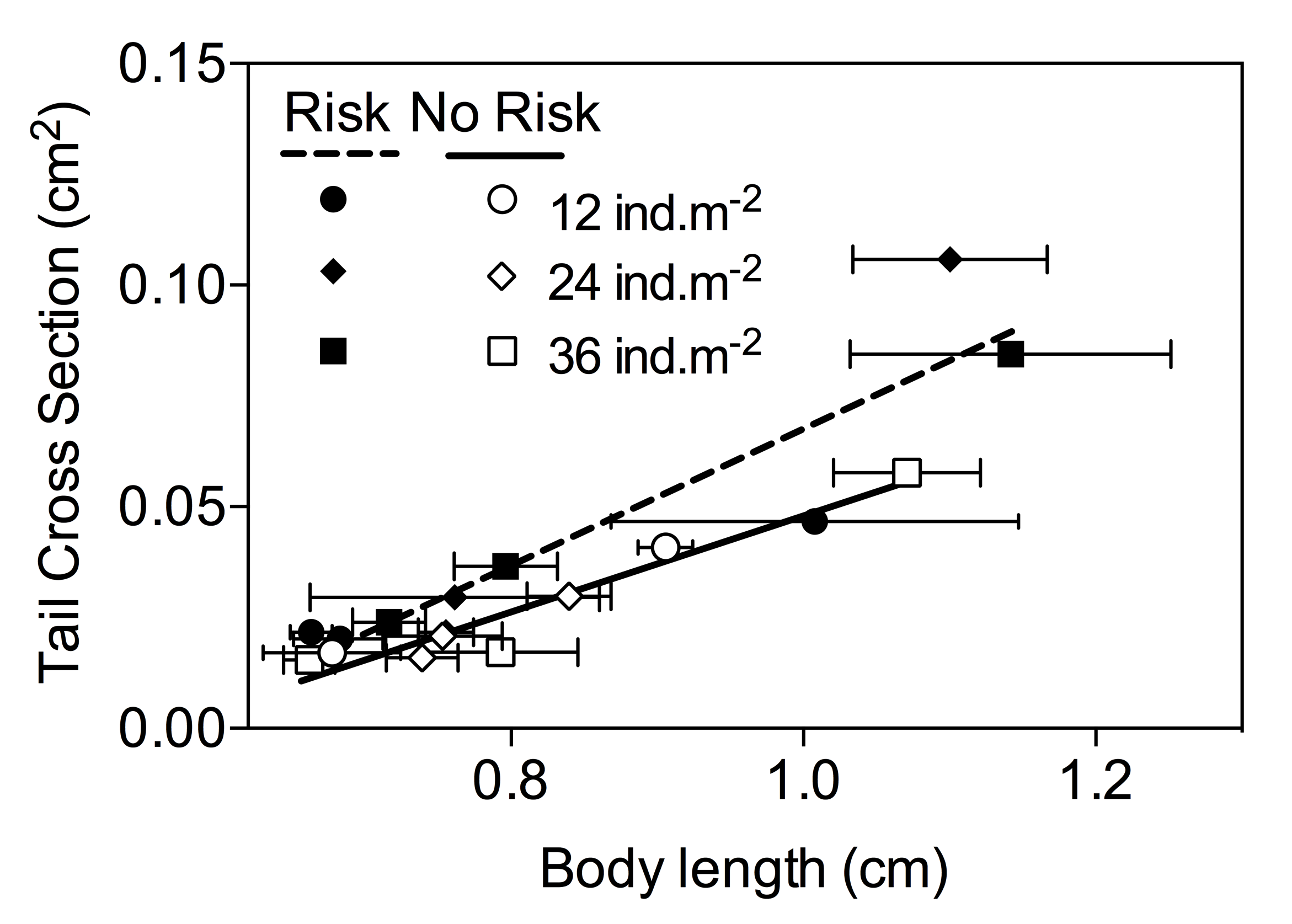

Supplement: Supplementary file 1 — Appendix S1. (A) Tadpoles body nutrient content (right panels) and excretion rates (left panels) regressed against conspecific density at the end of the experiment in the presence and absence of risk predation cues. (B) Comparison of N (Nitrogen) and P (phosphorus) concentrations and N:P ratio (mean ± SD) in water column among treatments at the beginning of the experiment. (C) Loadings (i.e., eigenvalues) and percentage of variation in data for principal components analysis of the three measures of tadpole body and excretion stoichiometry. (D) A proxy of muscle mass was inferred from the product between tail muscle width and depth, which represents a cross section of the tail muscle. [file ECE3-5-4747-s001.docx]
